# Psychological Treatment of Co‐Occurring Trauma History, Posttraumatic Stress Disorder, and Eating Disorders: A Systematic Review of Clinical Outcomes

**DOI:** 10.1002/erv.3195

**Published:** 2025-04-10

**Authors:** Rachel E. Liebman, Kristen M. Hernandez, Jennifer Ip, Jessica Burdo, Kathryn Trottier

**Affiliations:** ^1^ Centre for Mental Health University Health Network Toronto Canada; ^2^ Department of Psychiatry University of Toronto Toronto Canada; ^3^ Department of Psychology Toronto Metropolitan University Toronto Canada

## Abstract

**Objective:**

The association between eating disorders (EDs) and both trauma exposure and posttraumatic stress disorder (PTSD) is well established. Existing evidence is inconclusive about the impact of trauma exposure and/or comorbid PTSD on ED treatment outcomes and if comorbid ED affects PTSD treatment outcomes. The objective of this systematic review is to consolidate the literature on treatment efficacy and effectiveness for individuals with EDs and trauma histories with and without PTSD, as well as to understand how ED and PTSD symptoms may impact improvement in each other, and how improvements in ED and PTSD symptoms might differ across treatment focus (ED‐focused, PTSD‐focused or both) and modality (e.g., cognitive behavioural, psychodynamic, transdiagnostic, integrative).

**Methods:**

A systematic search of peer‐reviewed publications was conducted across three databases (PsycINFO, PubMed, SCOPUS).

**Results:**

Results indicate that comorbid PTSD symptoms, but not trauma history alone, may negatively affect ED treatment outcomes. Likewise comorbid ED symptoms may negatively affect PTSD treatment outcomes, but data is lacking. ED and/or PTSD symptom improvement was observed across treatment modalities.

**Conclusions:**

Results suggest that individuals may respond to a variety of modalities. Integrated and concurrent treatments show promise as an effective strategy to achieve long‐term recovery from this debilitating comorbid condition.

**Summary**

- Trauma history is not associated with ED treatment outcomes.
- Comorbid PTSD symptoms are negatively associated with ED treatment outcomes.
- Comorbid ED symptoms are negatively associated with PTSD treatment outcomes.
- People with EDs, trauma histories and PTSD improve in multiple treatment modalities.
- Integrated and concurrent treatments may promote more sustained recovery for ED‐PTSD.

## 1 Introduction

Research demonstrates that co‐occurrence of eating disorders (EDs) with trauma exposure (TE) and posttraumatic stress disorder (PTSD) symptoms may impede improvements in treatment for both conditions. Trauma exposure is a nonspecific risk factor for ED development (Molendijk et al. 2017; Mendoza et al. 2024). PTSD is one common outcome of trauma exposure and accumulating research has demonstrated a unique association between ED and PTSD symptoms beyond what can be explained by trauma exposure alone (Longo et al. 2024). Estimates of TE among individuals seeking treatment for an ED range from 18% to 80% (Backholm et al. 2013; Kjaersdam Telléus et al. 2021; Mitchell, Mazzeo, et al. 2012) and an estimated 20% of individuals diagnosed with an ED also meet criteria for PTSD (Ferrell et al. 2022). Trauma exposure and/or PTSD (TE/PTSD) are associated with a higher rate of drop out, lower likelihood of remission at end of treatment (EOT) or follow‐up, and higher likelihood of relapse in EDs (Day et al. 2024; Convertino and Mendoza 2023). In contrast, little is known about whether ED symptoms impact PTSD treatment outcomes. Greater understanding of how the combined presentation of TE/PTSD and EDs impact treatment response for both conditions would inform clinical decision‐making and improve treatment development.

### 1.1 The Functional Model of PTSD and ED Symptoms

Researchers have pointed to a potentially reinforcing functional relationship in which trauma exposed individuals use food restriction, binge eating, and/or purging as a means of avoiding trauma related thoughts and memories (e.g., Liebman et al. 2021; Trottier et al. 2016; Mitchell, Scioli, et al. 2021). This avoidance prevents trauma‐related thoughts and emotions from being processed, which sustains these symptoms (e.g., intrusive memories and hyperarousal) while normalising ED‐related behaviours as coping mechanisms. Left untreated, this functional relationship may keep both disorders active and contribute to relapse. Multiple studies using mediational, network, and ecological momentary analyses (e.g., Sandhu et al. 2023; Liebman et al. 2021; Vanzhula et al. 2019; Nelson et al. 2022; Karr et al. 2013) support this premise by offering evidence of bidirectional associations between PTSD and ED. They underscore that ED behaviours may be a reinforcing method of managing PTSD that may contribute to relapse for both conditions.

### 1.2 Previous Systematic Reviews on Co‐Occurring Trauma History, PTSD and EDs

Two recent systematic reviews examined ED treatment outcomes of individuals with EDs and TE/PTSD (Convertino and Mendoza 2023; Day et al. 2024). Both reviews had mixed findings but broadly concluded that individuals with ED and TE showed similar improvements in ED symptoms relative to individuals with EDs and no TE, but were more likely to drop out from treatment or relapse after treatment ended. Both reviews also found mixed evidence that PTSD moderates ED symptom improvement and highlighted the paucity of research investigating this relationship.

These reviews take an important step in advancing clinical understanding of how TE/PTSD may impact the course and outcome of ED treatment. However, a wider synthesis of the literature may prove productive for several reasons. First, both reviews are limited by the fact that they selected studies which included PTSD *diagnosis* as a predictor or moderator of treatment. Many individuals with EDs have subthreshold versus full syndrome PTSD because, for instance, PTSD symptoms are suppressed by the use of ED‐based avoidance strategies (Mitchell, Scioli, et al. 2021). This criterion is likely to exclude many individuals with significant ED‐PTSD psychopathology and symptomatology, which may skew findings. Examining PTSD symptoms continuously may provide a more accurate picture of the potential impact of ED‐PTSD comorbidity on treatment outcomes. Second, existing reviews only examined the effect of PTSD as a predictor or moderator of ED treatment outcomes and not the potential impact of ED symptoms on PTSD treatment outcomes. A functional relationship between ED and PTSD implies a bidirectional effect such that ED symptoms would also interfere with PTSD improvement. Finally, the reviews did not examine potential differences in outcomes across treatment modalities. Many clinical programs use alternative modalities (e.g., psychodynamic, integrative, transdiagnostic) or adjunctive components (e.g., neurofeedback). Parsing out how effects align or differ across treatment modalities may elucidate reasons for mixed findings in previous reviews and inform treatment development.

### 1.3 The Current Review

The objective of this systematic review is threefold. Our first aim was to examine the efficacy and effectiveness of existing psychotherapy treatments for individuals with TE/PTSD and ED symptoms. This included the primary outcome of change in severity of ED and/or PTSD symptoms, as well as secondary outcomes including rates of ED or PTSD remission, relapse, and treatment drop‐out, and other concomitant clinical outcomes (e.g., depression, anxiety, general psychopathology). Our second aim was to examine to what extent PTSD symptoms and ED symptoms predict or moderate improvements in each other. Our third aim was to expand on past reviews with respect to how improvements in ED and PTSD symptoms might differ across clinical focus of treatment (e.g., ED‐focused, trauma‐focused, concurrent/integrated treatments) and psychotherapeutic modalities. We also provide recommendations for future research.

## 2 Methods

### 2.1 Study Selection Criteria

A systematic search of peer‐reviewed publications was conducted across three databases (PsycINFO, PubMed, SCOPUS) in accordance with the Preferred Reporting Items for Systematic Reviews and Meta‐Analysis (PRISMA) statement (Moher et al. 2009) in April 2024. The search strategy and full inclusion/exclusion criteria are shown in Table 1. The search was restricted to title/abstract/keywords in SCOPUS. No limitations were placed on publication date, participant age, or treatment setting or format. Both published and unpublished studies were included. The first three authors (R.E.L., K.H., and J.I.) screened the articles for eligibility based on title, abstract, and full‐text review screening with 91% agreement between the three raters.

**TABLE 1:** Search strategy, inclusion criteria and exclusion criteria for study selection.

Search strategy	‘eating disorder’ OR anorexi* OR bulimi* OR ‘binge eating disorder’ OR ‘other specified feeding and eating disorder’ OR ‘OSFED’ OR ‘atypical eating disorder’ OR ‘purging disorder’ OR ‘avoidant/restrictive food intake disorder’ OR ‘avoidant restrictive food intake disorder’ OR ARFID OR ‘disordered eating’ AND trauma* OR abus* OR ‘posttraumatic stress’ OR ‘post‐traumatic stress’ OR ‘post traumatic stress’ OR ‘PTSD’ AND therap* OR psychotherap* OR counsel* OR treatment OR intervention
Inclusion criteria	Empirical studies or case series that: (1) reported aggregated outcomes with at least 10 participants; (2) were published in English language, (3) delivered a psychological treatment; (4) assessed change in symptoms; and (5) examined the relationship between ED symptoms or diagnosis and either trauma exposure or PTSD symptoms or diagnosis
	The relationship between ED and trauma history and/or PTSD could have been examined in 2 ways: (5a) ED symptoms/diagnosis, trauma history and/or PTSD symptoms/diagnosis as a **predictor or moderator** *of either* ED symptoms/diagnosis or PTSD symptoms/diagnosis or (5b) both ED and PTSD symptoms/diagnosis as treatment **outcome variables**
Exclusion criteria	Pharmacological and biofeedback treatments unless offered in conjunction with psychotherapy; qualitative studies; review articles; study protocols; and case studies

### 2.2 Assessment of Methodological Quality of Selected Studies

Two raters (R.E.L. and J.B.) assessed the methodological quality of included studies with the 27 item Downs and Black Checklist (Downs and Black 1998). Scores range from 0 to 28. Quality levels range from excellent (26–28); good (20–25); fair (15–19); to poor (≤ 14) (Downs and Black 1998). Test‐retest reliability, inter‐rater reliability, and internal consistency of this instrument are all good (Downs and Black 1998). Raters discussed discrepancies to reach consensus. Initial percent agreement before discrepancies were addressed was 91%.

## 3 Results

**FIGURE 1:**
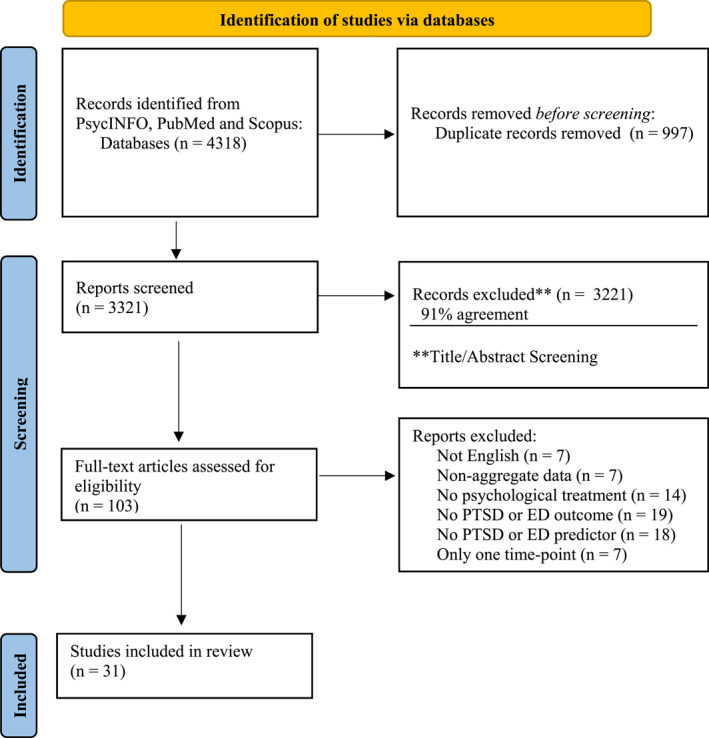
PRISMA diagram of systematic review search and reasons for exclusion.

The search across databases resulted in a total of 3321 identified studies, of which 137 were selected for full text screening. After screening, 31 articles met inclusion criteria (see Table 2). Figure 1 presents a flow diagram of the screening and selection process.

**TABLE 2:** Sample and treatment characteristics of included studies.

Study	Design, *N*, location	Demographics	# Sessions	Therapy format(s); therapy type	Sample
		*M* (SD) or %			
Studies examining trauma exposure and ED					
Anderson et al. (1997)	Naturalistic, 76, USA	Age = 27 (9.3); 100% f, 89% White	Variable	Inpatient (Ind., group, nutrition; ‘behaviorally oriented’)	DSM‐III‐R BN
Billman Miller et al. (2024)	Chart review, 154, USA	Age NR for full sample; 92.2% f; 94% White	NR for full sample	Partial (Ind., group, family, meal support; CBT)	DSM‐5 AN, Atypical AN
Cabelguen et al. (2023)	Naturalistic, 405, France	Age = 26.5 (9.9); 96% f; race/ethnicity NR	NR	Outpatient and inpatient (multi‐professional care; biopsychosocial)	DSM‐IV/5 AN‐BP, BN, BED
Calugi et al. (2018)	Naturalistic, 81, Italy	Age = 23.6 (6.2); 96.3% f; race/ethnicity NR	20 weeks	Inpatient and partial (Ind., group, meal support; CBT‐E)	DSM‐5 AN
Cassioli et al. (2022)	Naturalistic, 120, Italy	Age = 25.2 (9.6); 100% f; race/ethnicity NR	1 year	Outpatient (Ind.; CBT‐E)	DSM‐5 AN
Castellini et al. (2018)	Naturalistic, 133, Italy	Age NR for full sample; 94.7% f; 100% White	19–40	Outpatient (Ind.; CBT‐E)	DSM‐IV AN, BN
Kopland et al. (2023)	RCT, 130, Norway	Age = 30.9 (9.7); 97% f; race/ethnicity NR	13 weeks	Inpatient (Ind. and group; CBT versus CFT)	DSM‐IV/5 AN, BN, OSFED
Lelli et al. (2019)	Naturalistic, 69, Italy	Age NR for full sample; NR % f; 100% White	19–40	Outpatient (Ind.; CBT)	DSM‐IV AN, BN
Ridley (2009)	Naturalistic, 398, USA	Age = 22 (7.5); 100% f; 96.5% White, 0.6% Black, 0.3% Hispanic, 0.6% Asian	NR	Inpatient (Ind., group, family, dietary support)	DSM‐IV AN, BN, EDNOS
Rossi et al. (2024)	Multiple baseline, 75, Italy	Age NR for full sample; 100% f, race/ethnicity NR	60–65	Outpatient (Ind., dietary support; CBT vs. CBT + EMDR)	DSM‐5 AN
Serra et al. (2020)	Naturalistic, 142, Belgium	Age = 38.7 (10.8); 88% f; NR race/ethnicity	6 months	Outpatient (group; CBT‐E)	DSM‐5 BED
Strangio et al. (2017)	Naturalistic, 26, Italy	Age range = 13–18; 61.5% f; race/ethnicity NR	12 months	Partial (Ind.; psychodynamic)	DSM‐IV EDs
Vrabel et al. (2010)	Naturalistic, 74, USA	Age = 2 9 (7.3); 98.8% f; NR race/ethnicity	15–23 weeks	Inpatient (Ind., group, family; CBT)	DSM‐IV AN, BN, EDNOS
Vrabel et al. (2024)	RCT, 130, Norway	Age = 30.9 (9.7); 97.7% f; 97.6% White, 0.01% African, 0.02% Latino	13 weeks	Inpatient (ind., group; CFT vs. CBT‐E)	DSM‐IV/5 AN, BN, OSFED
Wolfe et al. (2009)	Naturalistic, 116, United Kingdom	Age = 26.8 (8.8); NR % f; 100% White	*M* = 169.74 (142.7) days	Partial (Ind., group, family, meal support; CBT)	DSM‐IV AN, BN, EDNOS
Studies examining PTSD and ED					
Brewerton et al. (2023)	Naturalistic, 609, USA	Age = 26 (8.8); 95.8% f; 93.1% White, 3.3% Asian, 2.2% Black, 1.1% AI/NA, 0.4% PI	NR	Residential (Ind., nutrition, family, yoga; CPT)	DSM‐5 AN, BN, OSFED
Brewerton et al. (2024)	Naturalistic, 18, USA	Age = 32.6 (11.8); 77.8% f; 83.3% White, 5.6% Black, 11.1% NA	19–125 days	Residential (Ind., nutrition, family, yoga; CPT)	DSM‐5 AN, BN, OSFED
Claudat et al. (2022)	Chart review, 57, USA	Demographics NR for full sample	*M* = 10.40 (5.13)	Partial (group, Ind; PE, CPT)	DSM‐5 ED + PTSD
Christoffersen et al. (2024)	Naturalistic, 36, Norway	Age = 32.6 (9.1); 100% f, race/ethnicity NR	13 weeks	Inpatient (Ind., group, meal support, family; CFT)	DSM‐IV AN, BN, EDNOS
Cohen et al. (2010)	RCT, 122, USA	Age = 38 (10.0); 100% f; 42.6% White, 32.8% AA, 9.8% Latina, 14.8% other	12	Outpatient (group; SS vs. women's health education)	DSM‐IV PTSD + SUD
Hazzard et al. (2021)	RCT, 112, USA	Age = 39.7 (13.4); 82.1% f; 92% White	ICAT: 21, CBT: 10	Outpatient (Ind.; ICAT vs. guided self‐help CBT‐E)	DSM‐5 BED
Mitchell, Mazzeo, et al. (2012) and Mitchell, Wells, et al. (2012)	RCT dismantling, 65, USA	Age = 35.4 (12.4); 100% f; 62% White 34% AA, 0.7% Asian, 1.3% AI, 2% other	12	Outpatient (Ind.; CPT)	DSM‐IV PTSD
Mitchell, Scioli, et al. (2021) and Mitchell, Singh, et al. (2021)	Naturalistic, 2809, USA	Age = 25.1 (11.0); 100% f; 80.5% White, 2.1% Black, 0.5% NA, 2.3% Asian/PI, 2.2% other, 3.5% Multiracial	*M* = 32.1 (14.1) days	Residential (group, ind.; nutrition; UTM)	DSM‐IV/5 EDs
Pratt (2004)	RCT, 69, USA	Age = 37.3 (8.8); 100% f; 73.9% White, 15.9% Black, 7.3% Hispanic, 2.9% other	12	Outpatient (group; SS vs. TAU)	DSM‐IV PTSD + SUD
Rienecke et al. (2021)	Naturalistic, 613, USA	Age = 24.5 (9.8); 83.8% f; 71.1% White, 3.8% mixed race, 3.4% Hispanic, 2% Asian, 0.8% Black, 0.2% NA	*M* = 8.86 (5.58) weeks	Inpatient, residential, or partial (Ind., group, family; meal support; ‘trauma‐informed’)	DSM‐5 EDs
Rodriguez et al. (2005)	Naturalistic, 270, Columbia	Age = 21.4 (7.21); 100% f; race/ethnicity NR	4 months as study end	Outpatient (Ind., group, family, and nutrition; CBT, psychodynamic)	DSM‐IV AN, BN, BED
Scharff et al. (2021)	Naturalistic, 1055, USA	Age = 24.7 (10.7); 100% f; > 80% White	*M* = 33.28 days	Residential (Ind., group, meal support; UTM)	DSM‐5 EDs
ten Napel‐Schutz et al. (2022)	Multiple baseline, 12, Netherlands	Age = 26.4 (12.0); 100% f; race/ethnicity NR	12	Partial (Ind., group, meal support; CBT, family; IR)	DSM‐5 AN + PTSD
Trottier et al. (2017)	UCT, 11, Canada	Age = 30.3 (7.6); 100% f; > 80% White	16	Outpatient (Ind., CBT‐ED‐PTSD)	DSM‐5 ED‐PTSD
Trottier et al. (2022)	RCT, 43, Canada	Age = 29.2 (9.2); 97.6% f; > 80% White	16	Outpatient (Ind.; CBT‐ED vs. CBT‐ED‐PTSD)	DSM‐5 ED‐PTSD
Winkeler et al. (2022)	RCT, 36, Germany	Age = 28.4 (5.9); NR % f; NR race/ethnicity	12	Inpatient (Neurofeedback vs. ‘media relaxation’, ind., group, nutrition)	ICD‐10 ED + PTSD

Abbreviations: AI = Alaskan Indian, AN‐BP = anorexia nervosa‐binging/purging subtype, BED = binge eating disorder, BN = bulimia nervosa, CBT‐E = cognitive behaviour therapy‐enhanced, CFT = compassion focused therapy, CPT = cognitive processing therapy, DSM = diagnostic and statistical manual, Dx = diagnosis, ED = eating disorder, EDNOS = eating disorder not otherwise specified, EMDR = eye movement desensitisation and reprocessing, F = female, ICAT = integrative cognitive‐affective therapy, ICD‐10 = International Classification of Diseases‐10, Ind. = individual, IR = imagery rescripting, NA = Native American, NR = not reported, OSFED = other specified feeding or eating disorder, PE = prolonged exposure, PI = Pacific Islander, PTSD = posttraumatic stress disorder, RCT = randomized clinical trial, SS = Seeking Safety, SUD = substance use disorder, Sx = symptoms, TAU = treatment as usual, UCT = uncontrolled clinical trial, UTM = Unified Treatment Model.

### 3.1 Sample Characteristics of Included Studies

The sample characteristics of the included studies are presented in Table 2. Sample sizes ranged from 10 (ten Napel‐Schutz et al. 2022) to 2809 (Mitchell, Singh, et al. 2021). Twenty‐four studies used adult samples and seven used adolescent only or adolescent and adult samples. The majority of studies (*n* = 22) involved entirely or almost entirely (> 90%) female samples. Fourteen studies had primarily (> 80%) White samples, and 13 did not report on race or ethnicity. Nineteen studies were conducted in an intensive treatment programme (i.e., inpatient, residential, or partial hospitalisation) and 12 in an outpatient programme. Eight examined an individual therapy, three examined group therapies and 19 were a combination of individual, group, and/or family therapy within a multimodal treatment programme.^1^

### 3.2 Methodological Quality of Selected Studies

Table 3 presents quality ratings of the included studies. Ratings ranged from 12 (Brewerton et al. 2024) to 25 (Trottier et al. 2022), indicating poor to good study quality. The majority of studies were uncontrolled or consecutive naturalistic designs. Eight studies were randomized controlled trials (RCTs) with an active or placebo control group. All studies described the hypotheses and exploratory findings thoroughly, had a representative setting, and used appropriate statistics and outcome measures. A minority of studies conducted a priori power calculations, concealed randomisation, reported adverse events, or described intervention compliance.

### 3.3 Treatment Outcomes

Below, we describe treatment outcome findings from the included studies. Studies are organised first by predictor (TE, PTSD, or ED), then by focus of the treatment (ED‐focused, PTSD‐focused, or integrative/concurrent), and finally by treatment modality. Key findings of each study are summarised in Table 3 along with descriptions of key predictor and outcome measures.

**TABLE 3:** Predictors, outcomes and results of included studies.

Study	Summary of results	Predictor and primary measures	QR
Studies examining trauma exposure and ED			
Anderson et al. (1997)	Both abused and non‐abused women significantly improved in ED sx and bxs by EOT. Abuse status or severity did not moderate improvement. The abused group had clinically significant EOT sx/bx, less full remission, and more rehospitalisation at FU	P: Child sexual abuse (clinical interview)	17
		O: ED sx (EDI‐2; nurse‐reported ED bxs)	
Billman Miller et al. (2024)	Childhood abuse did not predict change in global ED sx at EOT, but emotional abuse predicted less improvement in discharge shape and weight overvaluation	P: Lifetime trauma (chart review)	20
		O: ED sx (EDE‐Q), BMI	
Cabelguen et al. (2023)	History of childhood abuse did not predict ED sx at 1 year	P: Child abuse (EVE)	18
		O: ED dx (MINI)	
Calugi et al. (2018)	Both abused and non‐abused patients improved in ED sx. Abuse status did not predict ED outcomes at EOT or FU	P: Sexual abuse before ED onset (clinical interview)	21
		O: ED sx (EDE‐Q)	
Cassioli et al. (2022)	Childhood TE predicted less ED sx improvement	P: Child abuse (CTQ)	18
		O: ED sx (EDE‐Q)	
Castellini et al. (2018)	No baseline differences in ED psychopathology between patients with versus without childhood abuse. Childhood TE predicted higher likelihood of rehospitalisation and drop out from treatment but did not predict full remission of ED diagnosis at 3‐year FU. Drop out was more common in patients who experienced both abuse and neglect	P: Child abuse (CEAQ)	21
		O: ED sx (EDE‐Q) and body uneasiness (BUT)	
Kopland et al. (2023)	For patients with childhood trauma in the CFT‐E group, self‐compassion predicted ED sx improvement, but there was no relationship between self‐compassion and ED sx improvement for non‐traumatised patients	P: Child abuse (CTQ)	23
		O: ED sx (EDE‐Q)	
Lelli et al. (2019)	All patients showed reductions in ED sx by EOT except those with BN and child abuse. Child abuse did not predict ED remission. Patients without child abuse showed significant improvements in general psychopathology, but patients with child abuse did not	P: Child abuse (CEAQ)	15
		O: ED sx (EDE‐Q)	
Ridley (2009)	CSA predicted greater improvement in ED sx than non‐CSA	P: Child sexual abuse (LES, clinical interview)	15
		O: ED sx (EAT)	
Rossi et al. (2024)	Patients with child abuse did not improve in BMI, general and ED psychopathology or dissociation after 40 sessions of CBT‐E, but did improve after a second course of CBT‐E or CBT+EMDR. Those receiving CBT+EMDR showed greater improvements. Only those receiving CBT+EMDR improved in general psychopathology and dissociative sx. For traumatised patients, reduction in ED sx was mediated by reduction in dissociation	P: Child abuse (CTQ)	22
		O: ED sx (EDE‐Q)	
Serra et al. (2020)	Number of TE did not predict improvement or remission from binge eating episodes. Perceived impact of the trauma negatively predicted remission from binge eating at ED, and this effect was partially mediated by dissociative sx	P: Presence and impact of lifetime trauma (TEC)	18
		O: Binges per week	
Strangio et al. (2017)	Childhood TE did not predict improvement in ED sx	P: Child abuse (CTQ)	13
		O: ED sx (EDI‐3)	
Vrabel et al. (2010)	CSA predicted less improvement in global ED sx by EOT	P: Child sexual abuse (chart review)	19
		O: ED sx (EDE)	
Vrabel et al. (2024)	Changes in ED sx did not significantly differ between CFT‐E and CBT by TE. CFT‐E resulted in greater ED sx improvements than CBT at 1‐year FU for TE, but not non‐TE, participants. All participants in CFT‐E significantly improved in general psychopathology and non‐TE (but not TE) participants had significantly improved PTSD sx at EOT. In the CBT condition, TE participants significantly improved in interpersonal problems	P: Child abuse (CTQ)	24
		O: ED sx (EDE); PTSD sx (PSS‐SR)	
Wolfe et al. (2009)	Absence of CSA predicted decrease in anorexic and bulimic cognitions. Childhood abuse status did not predict improvement in clinician‐rated ED symptoms	P: Lifetime abuse, death, divorce (chart review)	17
		O: ED sx (SEDS); ED sx (clinician rated)	
Studies examining PTSD and ED			
Brewerton et al. (2023)	Participants with and without PTSD had statistically significant improvement in ED, PTSD, depression, anxiety, and ED QOL at 6‐month FU. Participants with PTSD reported higher ED, depression, and anxiety, and worse QOL at all timepoints. They showed non‐significantly greater improvement in ED sx and ED‐related QOL during treatment than those without PTSD. Significantly fewer patients with versus without PTSD achieved remission in ED, PTSD, depression, and state‐trait anxiety at EOT and 6‐month FU	P: PTSD dx (LEC, PCL‐5)	19
		O: ED dx and sx (EDE‐Q, EDI‐2)	
Brewerton et al. (2024)	Patients with ED+DID demonstrated statistically significant improvements in self‐reported ED, PTSD, depression, anxiety, and QOL at EOT	P: PTSD dx (LEC, PCL‐5)	12
		O: ED dx and sx (EDE‐Q, EDI‐2)	
Claudat et al. (2022)	Participants with more ED sx and less ED‐related psychosocial impairment showed greater reductions in PTSD sx at EOT. Participants who received CPT or PE experienced statistically significant improvement in their PTSD sx and statistically significant and clinically meaningful changes in ED sx, ED‐related psychosocial impairment, and anxiety	P: PTSD dx (MINI or SCID‐5) and sx (PCL‐5)	17
		O: ED sx (EDE‐Q)	
Christoffersen et al. (2024)	Participants demonstrated significant, medium‐effect size decreases in ED sx at end of individual and group CFT‐E, but no significant change in trauma sx or self‐compassion	P: Child abuse (CTQ)	19
		O: ED sx (EDE‐Q); PTSD sx (PSS‐SR)	
Cohen et al. (2010)	PTSD improved for participants with and without binge eating. Women who endorsed binge eating showed significantly less PTSD improvement and abstinence from substance use over the course of treatment and 3‐, 6‐, and 12‐month FUs. There was no difference in sx improvement by treatment condition for women who did versus did not binge eat	P: PTSD sx (CAPS‐IV, PSS‐SR)	18
		O: Binges Y/N; (EDE‐Q)	
Hazzard et al. (2021)	Child abuse and lifetime PTSD predicted less improvement in OBE frequency but not ED psychopathology at EOT and 6‐month FU. Lifetime PTSD moderated relationship between child abuse and OBE frequency at 6‐month FU. Child abuse predicted greater OBE frequency among people with, but not without, lifetime PTSD. Neither PTSD nor the interaction between child abuse and PTSD predicted ED psychopathology at EOT or 6‐month FU. There was no differences between treatment conditions	P: PTSD dx (SCID‐IV), child abuse (CTQ)	19
		O: ED sx (EDE)	
Mitchell, Mazzeo, et al. (2012) and Mitchell, Wells, et al. (2012)	Participants showed mean‐level decreases in body dissatisfaction but not ED‐specific outcomes during CPT. Improvements in PTSD sx were significantly associated with decreases in characteristics common to both PTSD and ED (e.g., interoceptive awareness, interpersonal distrust, impulse regulation, ineffectiveness, and maturity fears). PTSD sx improvement was not associated with changes in body dissatisfaction	P: PTSD dx (CAPS ‐IV) and PTSD sx (PDS)	18
		O: ED sx (EDI‐2)	
Mitchell, Scioli, et al. (2021) and Mitchell, Singh, et al. (2021)	There was no difference in ED symptom change or likelihood of treatment drop out by end of treatment or 6‐month FU between participants with versus without a PTSD diagnosis	P: Lifetime assault (clinical interview)	18
		PTSD dx (CAPS‐IV), PTSD sx (PDS)	
		O: ED sx (EDE‐Q)	
Pratt (2004)	Baseline ED sx predicted less trauma‐related sx improvement at 6‐month FU, but not EOT or 12‐month FU. ED sx did not predict substance use improvement at EOT or either FU. Baseline trauma‐related sx predicted less improvement in ED sx at EOT, as well as 6‐ and 12‐month FU, and there was no effect of treatment condition on ED sx improvement	P: Trauma history (THQ); PTSD dx (SCID‐IV) and PTSD sx (TSC‐40)	20
		O: ED sx (EDI‐2)	
Rienecke et al. (2021)	Participants had significant reductions in self‐reported ED and PTSD sx by EOT. Those with PTSD had medium‐to‐large effect size improvements in PTSD over treatment, and those with subthreshold PTSD had small effect size improvements. A significantly smaller percentage of patients met criteria for full threshold PTSD at EOT versus baseline	O: ED sx (EPSI) PTSD dx and sx (PCL‐5)	14
Rodriguez et al. (2005)	TE was associated with less ED sx improvement by 4 months of treatment. Cumulative sexual and violent TE prior to ED onset predicted greater likelihood of being categorised as a ‘poor responder’ to treatment and greater drop out by EOT. 20% of treatment non‐responders versus 10% of treatment responders, had a PTSD diagnosis	P: Abuse, violence before ED (clinical interview) and PTSD dx (SCID‐IV)	19
		O: ED sx (YBC‐EDS)	
Scharff et al. (2021)	Individuals with PTSD had more pronounced *improvement* in ED, experiential avoidance, and depression (but not anxiety, anxiety sensitivity, or mindfulness) sx by EOT, but greater deceleration in improvement and more sx recurrence from by 6‐month FU. They were less likely to have ED sx below clinical levels than those without a PTSD diagnosis	P: PTSD dx (unspecified semi‐structured interview)	18
		O: ED sx (EDE‐Q)	
ten Napel‐Schutz et al. (2022)	Participants had significant improvements in BMI and a large effect size reduction in dysfunctional body beliefs between by EOT. ED sx, PTSD sx, posttraumatic cognitions, and core negative emotions significantly improved by 3‐month FU with a large effect	O: PTSD sx (PSS‐SR); ED sx (EDE‐Q)	20
Trottier et al. (2017)	All participants had reliable improvements in clinician‐rated PTSD and 82% in self‐reported PTSD. There was no significant change in clinician‐rated ED psychopathology but ∼30% of participants experienced reliable improvement and 9% reliable worsening	O: ED sx (EDE‐Q); PTSD sx (CAPS‐5, PCL‐5)	16
Trottier et al. (2022)	Participants in the integrated treatment had significantly greater reductions in clinician‐ and self‐reported PTSD sx at EOT, 3‐, and 6‐month FU, and were more likely to remit from a PTSD diagnosis at EOT and FUs than those who received standard CBT‐ED. There was no significant difference in change in ED psychopathology between conditions	O: ED sx (EDE); PTSD sx (CAPS‐5, PCL‐5)	25
Winkeler et al. (2022)	Both the neurofeedback and the placebo control showed significant improvements in ED sx, BMI and PTSD hyperarousal, and marginal improvements in PTSD avoidance by EOT. There were significantly greater improvements in restrained eating and BMI and marginally greater improvements (*p* = 0.06) in avoidance in the neurofeedback condition	O: PTSD sx (IES‐R); ED sx (EDE‐Q), BMI	22

Abbreviations: BMI = body mass index, BUT = Body Uneasiness Scale, Bx = behaviours, CAPS = Clinician Administered PTSD Scale, CBT‐E = cognitive behaviour therapy‐enhanced, CEAQ = Childhood Emotional Abuse Questionnaire, CFT‐E = compassion‐focused therapy for eating disorders, CPT = Cognitive Processing Therapy, CTQ = Childhood Trauma Questionnaire, DID = Dissociative Identity Disorder, Dx = diagnosis, EAT = Eating Attitudes Test, EAT = Eating Attitudes Test, ED = eating disorder, EDE = Eating Disorder Examination‐Interview, EDE‐Q = Eating Disorder Examination‐Questionnaire, EDI = Eating Disorder Inventory, EMDR = Eye Movement Desensitisation and Reprocessing, EOT = end of treatment, EPSI = Eating Pathology Symptom Inventory, EVE = French Life Events Questionnaire, FU = follow up, IES‐R = Impact of Events Scale‐Revised., LES = Life Events Survey, MINI = Mini International Neuropsychiatric Interview, O = outcome, OBE = objective binge episode, P = predictor, PCL‐5 = PTSD checklist‐5, PDS = Posttraumatic Diagnostic Scale, PE = Prolonged Exposure, PSS‐SR = PTSD Symptom Scale‐Self‐Report, QOL = Quality of life, QR = quality rating, SCID = Structured Clinical Interview for DSM IV/5, SEDS = Stirling Eating Disorder Scale, Sx = symptoms, TE = trauma exposure, TEC = Traumatic Experiences Checklist, TSC‐40 = Trauma Symptom Checklist‐40, YBC‐EDS = Yale‐Brown‐Cornell Eating Disorders Scale.

#### 3.3.1 TE Predicting or Moderating Outcomes From Psychotherapies for ED

##### 3.3.1.1 Cognitive Behavioural Therapies for ED

In all five studies that examined the effect of childhood TE on outcomes from outpatient Enhanced Cognitive Behavioural Therapy (CBT‐E; Fairburn 2008), both TE and non‐TE participants experienced ED symptom improvement.

Three of four studies found differences based on history of TE. Castellini et al. (2018) found that over the course of treatment, individuals with childhood TE were more likely to be rehospitalised for their ED, had more ED diagnostic crossover, more DSM‐IV Axis I comorbidity, and more treatment drop out than those who had no history of childhood TE. At 3‐year follow‐up, ED remission rates did not differ by TE but those with TE had a higher persistence of mood and OCD symptoms than non‐traumatised peers. Lelli et al. (2019) did not find a significant difference in ED remission at EOT based on childhood TE but found that general psychopathology and cortisol response only improved in the non‐TE group. Serra et al. (2020) found that number of traumas did not predict ED symptom improvement (i.e., objective binge episodes per week) or remission of BED diagnosis at EOT but that higher perceived impact of the trauma significantly decreased the likelihood of remission at EOT, and this effect was partially mediated by dissociation. In contrast, Calugi et al. (2018) found no significant differences in dropout rates, general psychopathology, BMI, or work or social functioning based on abuse history.

##### 3.3.1.2 Multimodal Therapies for ED

All eight studies that examined TE as a predictor of ED outcomes in individual or group psychotherapy in a multimodal programme found that ED outcomes significantly improved for TE and non‐TE participants.

###### 3.3.1.2.1 Programs With CBT‐Only

All three studies that examined a CBT‐based multimodal outpatient, partial hospitalisation, or inpatient programme found that TE was associated with less improvement in ED outcomes at EOT (Wolfe et al. 2009; Billman Miller et al. 2024) and 5‐year follow up (Vrabel et al. 2010). Wolfe et al. (2009) found that history of childhood sexual abuse (CSA) was only associated with greater improvement in ED cognitions but not ED behaviours and Billman Miller et al. (2024) found that childhood abuse did not predict change in global ED symptoms at EOT, but history of emotional abuse specifically, was associated with less improvement in shape and weight overvaluation.

With regards to secondary outcomes, both studies found that CSA was not associated with change in BMI, but Wolfe et al. (2009) found that CSA was associated with less change in depression but not anxiety at the end of day treatment.

###### 3.3.1.2.2 Programs With Concurrent CBT and Other Modalities

Two studies examined a multimodal outpatient treatment programme. In an outpatient programme with concurrently delivered CBT and psychodynamic individual therapy, Rodriguez et al. (2005) found that TE was associated with less ED symptom improvement by 4 months of treatment (some individuals continued treatment beyond 4 months). Cumulative sexual and violent TE prior to ED onset predicted greater likelihood of being categorised as a ‘poor responder’ to treatment, defined as failure to meet specified markers for weight gain, ED behaviours, and binge/purge symptom control, and greater drop out by EOT.

Rossi et al. (2024) conducted a multiple baseline study of sequentially delivered outpatient CBT‐E followed by Eye Movement Desensitization and Reprocessing therapy (EMDR; Shapiro 2018) which was compared to a course of CBT‐E alone. Individuals who did not report histories of childhood abuse received 60‐65 sessions of CBT‐E. Participants with a history of childhood abuse were consecutively assigned to either this same course of CBT‐E or 40 sessions of CBT‐E followed by 20–25 sessions of EMDR. All participants were assessed after 40 sessions of CBT‐E (Time 1) and again after 20‐25 sessions of CBT‐E or EMDR (Time 2). TE participants in both the CBT‐E only and CBT‐E and EMDR conditions had no significant improvements in ED psychopathology or BMI at Time 1 but did at Time 2. Participants who received CBT‐E and EMDR reported greater improvements in ED psychopathology, and BMI at Time 2 than TE participants who only received CBT‐E. Those who received CBT‐E and EMDR also reported significant improvements in general psychopathology and dissociation at Time 2 while those who only received CBT‐E did not.

###### 3.3.1.2.3 Nonspecific Psychotherapies

Two of three studies found that a history of childhood physical abuse and/or CSA did not predict ED outcomes in a nonspecific psychotherapy as part of a multimodal outpatient treatment programme for EDs (Anderson et al. 1997; Cabelguen et al. 2023). In contrast, Ridley (2009) found that CSA history did predict improvements in ED symptoms in multiple levels of care. In this unpublished dissertation, individuals with a history of CSA had significantly *greater improvement* in self‐reported ED attitudes following ED services than those who did not have a history of CSA. Anderson et al. (1997) also found that full remission of ED behaviours at 3‐month follow up was less likely in the TE group and a significantly greater number of TE patients were rehospitalised for their ED at 3‐month follow up.

##### 3.3.1.3 Psychodynamic Psychotherapies for ED

Strangio et al. (2017) found that psychodynamic psychotherapy resulted in significant improvements in general psychopathology, interoceptive deficits, emotion dysregulation, and asceticism at EOT. Moreover, history of childhood abuse did not predict ED symptom improvement at EOT but there was less improvement in general psychopathology, impulsivity, and dissociation in those who experienced child abuse compared to those who did not.

##### 3.3.1.4 Compassion‐Focused Psychotherapies for ED Compared to CBT

Two studies compared individual and group CFT for EDs (CFT‐E; Goss and Allan 2014) to CBT for EDs (Waller et al. 2007). Kopland et al. (2023) conducted a RCT of individuals randomized to 13 weeks of CFT‐E or CBT for EDs and found that a history of childhood abuse did not predict improvement in ED symptoms at EOT. This study also found a significant three‐way interaction which indicated that within the CFT‐E group (but not the CBT group) there was a stronger relationship between changes in self‐compassion and changes in ED symptoms in those with childhood abuse histories than those without. Vrabel et al. (2024) conducted a long‐term follow‐up study of this sample and found that changes in ED symptoms from baseline to EOT did not significantly differ between the CFT‐E and CBT groups by TE and that CFT‐E resulted in greater ED symptom improvements than CBT at 1‐year postreatment for individuals with a history of childhood abuse (but not those without).

Vrabel et al. (2024) found significant improvements in the secondary outcome of general psychopathology for all participants in the CFT‐E condition, and significantly improved PTSD symptoms for the non‐TE (but not TE) participants in this condition at EOT. In the CBT condition, TE participants significantly improved in interpersonal problems.

##### 3.3.1.5 Integrative Psychotherapies for ED Compared to CBT

Hazzard et al. (2021) conducted an RCT of Integrative Cognitive Affective Therapy (ICAT; Wonderlich et al. 2015), adapted for BED compared to CBT guided self‐help (CBTgsh; Fairburn 2013) offered individually in an outpatient setting. Adjusting for baseline frequency of objective binge eating, moderate to severe childhood abuse predicted less improvement in objective binge eating frequency but not ED psychopathology at both EOT and 6‐month follow up. There was no difference in symptom improvement across treatment conditions.

#### 3.3.2 PTSD Predicting or Moderating ED Outcomes From Psychotherapies for ED

In all four studies that examined PTSD as a predictor or moderator of ED outcomes in an ED psychotherapy, ED symptoms improved over the course of treatment and/or follow up. Three of the four studies found that PTSD moderated at least one ED treatment outcome at EOT or follow‐up.

##### 3.3.2.1 Integrative Psychotherapies for ED Compared to CBT

Hazzard et al. (2021) found that lifetime PTSD predicted less improvement in objective binge episodes (but not ED psychopathology) at EOT and 6‐month follow up. In addition, lifetime PTSD moderated the association between childhood abuse and objective binge episode frequency at 6‐month follow up such that moderate/severe childhood abuse predicted greater objective binge episode frequency among participants with, but not without, a lifetime PTSD diagnosis. Neither PTSD nor the interaction between childhood abuse and PTSD predicted ED psychopathology at EOT or 6‐month follow up, and there was no difference in the effect of PTSD on ED outcomes between treatment conditions.

##### 3.3.2.2 Multimodal Therapies for ED

In their sample of *N* = 270 patients in their outpatient programme of concurrently delivered CBT and psychodynamic individual therapy, Rodriguez et al. (2005) found that 20% of treatment non‐responders versus 10% of treatment responders, had a PTSD diagnosis (*p* = 0.07) by 4 months of treatment.

##### 3.3.2.3 Transdiagnostic Psychotherapies for ED Compared to CBT

In two studies using quality improvement data from the same residential ED treatment programme, ED symptoms improved among participants receiving the Unified Treatment Model for EDs (UTM; Thompson‐Brenner et al. 2021) at EOT regardless of PTSD diagnostic status at admission. Using a sample of *N* = 1055 participants, Scharff et al. (2021) found that individuals with PTSD had more pronounced *improvement* in ED symptoms from baseline to EOT, but also greater deceleration in improvement and more symptom recurrence from baseline to 6‐month follow up, and they were less likely to have ED symptoms below clinical levels than those without a PTSD diagnosis. In contrast, using *N* = 2809 participants including the *n* = 1055 participants used in the Scharff et al. (2021) study, Mitchell, Singh, et al. (2021) found no difference in ED symptom change at EOT or 6‐month follow up between participants with versus without a PTSD diagnosis.

Scharff et al. (2021) also found that individuals with PTSD at admission improved more over the course of treatment on secondary outcomes of experiential avoidance and depression, but not anxiety, anxiety sensitivity or mindfulness, but, again these improvements did not sustain, showed more recurrence, and were less likely to reach a non‐clinical level by 6‐month follow up for individuals with versus without PTSD. Mitchell, Singh, et al. (2021) found no difference in treatment drop out at EOT or 6‐month follow up based on baseline PTSD status.

#### 3.3.3 ED Predicting or Moderating PTSD Outcomes From Psychotherapies for PTSD or PTSD‐SUD

No identified studies examined ED as a predictor or moderator of PTSD outcomes in a PTSD‐specific psychotherapy. In two studies of participants with comorbid PTSD and SUD randomized to Seeking Safety (SS), a 12‐session outpatient group CBT for comorbid PTSD and SUD (Najavits 2002), or treatment as usual (TAU), participants showed significant improvement in PTSD symptoms regardless of baseline ED symptoms. Participants did not need to endorse ED symptoms to be included in these studies. Pratt (2004) found that baseline ED symptoms predicted less trauma‐related symptom improvement at 6‐month follow up, but not EOT or 12‐month follow up. ED symptoms did not predict substance use improvement at EOT or either follow up. Cohen et al. (2010) found that adult women who endorsed binge eating and were randomized to either SS or a women's health education control condition showed significantly less PTSD improvement and less abstinence from substance use over the course of treatment and 3‐, 6‐, and 12‐month follow ups. There was no difference in symptom improvement by treatment condition for women who did versus did not binge eat.

#### 3.3.4 PTSD Predicting or Moderating ED Outcomes From Psychotherapies for PTSD‐SUD

Pratt (2004) also found that baseline trauma‐related symptoms predicted less improvement in ED symptoms at EOT, as well as 6‐ and 12‐month follow up, and there was no effect of treatment condition on ED symptom improvement.

#### 3.3.5 Association Between Changes in ED and PTSD From Psychotherapies for PTSD

One study by Mitchell, Wells, et al. (2012) examined bidirectional associations between changes in PTSD and changes in ED symptoms and characteristics in a randomized controlled dismantling trial of Cognitive Processing Therapy for PTSD (CPT; Resick et al. 2017). The study used a subsample of physically or sexually assaulted women who met criteria for PTSD and completed a measure of ED symptoms and characteristics. Participants did not need to endorse ED symptoms to be included in the study. Results showed mean‐level decreases in body dissatisfaction but not ED‐specific outcomes including bulimia, drive for thinness or non‐ED outcomes of perfectionism and social insecurity during CPT. Improvements in PTSD symptoms over the course of treatment were significantly associated with decreases in characteristics common to both PTSD and ED (e.g., interoceptive awareness, interpersonal distrust, impulse regulation, ineffectiveness, and maturity fears) but PTSD symptom improvement was not associated with changes in body dissatisfaction.

#### 3.3.6 ED Predicting or Moderating PTSD Outcomes From Concurrent ED‐PTSD Psychotherapies

Among individuals who were offered CPT or Prolonged Exposure (PE; Foa et al. 2007) in a partial DBT‐based hospitalisation programme (Claudat et al. 2022), those with more ED symptoms and less ED‐related psychosocial impairment showed greater reductions in PTSD symptoms at EOT.

#### 3.3.7 PTSD Predicting or Moderating ED Outcomes From Concurrent ED‐PTSD Psychotherapies

Brewerton et al. (2023) examined concurrent delivery of CPT to individuals in a multimodal residential ED treatment. Participants with PTSD reported higher ED, depression, and state‐trait anxiety symptoms and worse quality of life at all time points. Those with PTSD showed non‐significantly greater improvement in ED symptoms (*p* = 0.07) and ED‐related quality of life (*p* = 0.08) over the course of treatment than those without PTSD. Significantly fewer patients with PTSD achieved remission in ED, PTSD, depression, and state‐trait anxiety symptoms at discharge and 6‐month follow up than those without PTSD.

#### 3.3.8 ED and PTSD Outcomes From an Integrated or Concurrent Psychotherapy for ED‐PTSD

All six studies that examined a concurrent or integrated treatment for ED‐PTSD found that ED and PTSD symptoms sustained or improved over treatment and/or follow up.

##### 3.3.8.1 CPT Within a Multimodal ED Programme

Claudat et al. (2022) found that participants who received PTSD treatment experienced statistically significant improvement in their PTSD symptoms over the course of treatment and 70.2% achieved clinically meaningful change. There were also statistically significant and clinically meaningful changes in ED symptoms, ED‐related psychosocial impairment, and anxiety symptoms. Brewerton et al. (2023) found that participants with and without PTSD experienced statistically significant improvement in ED, PTSD, depression, anxiety, and ED quality of life over the course of treatment with medium‐to‐large effect sizes that were sustained at 6‐month follow‐up (with the exception of depression). Notably, the study did not account for whether or not patients received CPT as part of their treatment or how long the treatment lasted making it difficult to attribute positive changes to the PTSD treatment. Using a sample of 15 individuals from the same treatment facility who reported symptoms of dissociative identity disorder, Brewerton et al. (2024) found significant medium‐to‐large effect size improvements from admission to EOT in self‐reported ED and PTSD symptoms; depressive and state‐trait anxiety symptoms; and ED‐related quality of life.

##### 3.3.8.2 Imagery Rescripting (IMRS) Within a Multimodal ED Programme

ten Napel‐Schutz et al. (2022) conducted a multiple baseline case series of 10 underweight patients (BMI range = 14.6–18.4) with PTSD in an inpatient programme for EDs. Participants were provided concurrently delivered trauma processing using Imagery Rescripting (IMRS; Raabe et al. 2015). All participants were randomized to start IMRS anywhere from 3 to 7 weeks after beginning ED treatment. Results indicated significant improvements in BMI and a large effect size reduction in dysfunctional body beliefs between baseline and EOT. ED symptoms, PTSD symptoms, posttraumatic cognitions, and core negative emotions also significantly improved between baseline and 3‐month follow up with a large effect.

##### 3.3.8.3 Integrated CPT and CBT‐E Compared to CBT for ED

In an uncontrolled study, Trottier et al. (2017) delivered an integrated CBT treatment to patients with ED‐PTSD following a course of day treatment. Trottier et al. (2022) then conducted a RCT comparing the effects of the same integrated CBT for ED‐PTSD to standard CBT for ED following inpatient and/or day hospital ED treatment. The integrated treatment combined interventions from CBT‐E with CPT for PTSD. Both studies found significant improvements in PTSD and no significant changes in ED psychopathology. In Trottier et al. (2017), all participants had reliable improvements in clinician‐rated PTSD and 82% in self‐reported PTSD. There was no significant change in clinician‐rated ED psychopathology but approximately 30% of participants experienced a reliable improvement and 9% a reliable worsening. Trottier et al. (2022) found that those who received the integrated treatment experienced significantly greater reductions in both clinician‐rated and self‐reported PTSD symptoms at EOT, 3‐, and 6‐month follow up, and were more likely to remit from a PTSD diagnosis at EOT and follow‐ups than those who received standard CBT‐ED. There was also no significant difference in change in ED psychopathology between treatment conditions.

#### 3.3.9 ED and PTSD Outcomes From Other Novel or Adapted ED Treatments

All three studies that examined ED and PTSD outcomes from a novel or adapted ED treatment found significant improvements in ED symptoms and two (Rienecke et al. 2021; Winkeler et al. 2022) found significant improvements in PTSD symptoms at EOT.

Christoffersen et al. (2024) found significant, medium‐effect size decreases in ED symptoms at end of individual and group CFT‐E offered within a multimodal ED inpatient programme, but no significant change in trauma symptoms or self‐compassion. Rienecke et al. (2021) found evidence of improved ED‐PTSD symptoms in an intensive (inpatient, residential or partial hospitalisation) CBT‐based programme for EDs with a ‘trauma‐informed milieu’. The programme did not offer specific treatment for PTSD, but staff received training in trauma‐informed principles such as discussing traumatic events and building skills for managing trauma symptoms. Results indicated a significant reduction from baseline to EOT in self‐reported ED and PTSD symptoms (i.e., binge eating, purging, and food restriction) which significantly correlated with one another. Those with PTSD experienced medium‐to‐large effect size improvements in PTSD over treatment, and those with subthreshold PTSD exhibited small effect size improvements. A significantly smaller percentage of patients met criteria for full threshold PTSD at EOT compared to baseline. Finally, Winkeler et al. (2022) found evidence of improved ED‐PTSD symptoms in a RCT of infra‐low frequency neurofeedback compared to a ‘media‐supported relaxation’ placebo control offered to individuals with ED‐PTSD or ED‐subthreshold PTSD in their inpatient ED programme. Both the neurofeedback and the placebo control showed significant improvements in ED symptoms, BMI (for underweight patients) and PTSD hyperarousal and there was marginally significant improvement (*p* = 0.08) in PTSD avoidance from baseline to EOT. There were also significantly greater improvements in restrained eating and BMI, and marginally greater improvements (*p* = 0.06) in avoidance, in the neurofeedback than the placebo control condition.

## 4 Discussion

This review comprehensively examined the literature on existing psychotherapy treatments utilised with individuals who have TE/PTSD symptoms and ED symptoms. Our first aim was to examine the efficacy and effectiveness of the existing psychotherapy treatments that have been utilised with individuals who have both symptoms. Like past reviews (Day et al. 2024; Convertino and Mendoza 2023) the vast majority of studies that examined ED symptom improvements found that individuals with TE and/or PTSD experienced improvement in ED symptoms by EOT but had poorer maintenance of gains after treatment than individuals without TE and/or PTSD symptoms. Also echoing previous reviews, the majority of included studies concluded that TE did not moderate ED treatment outcomes but treatment response was poorer for individuals with TE with higher rates of ED remission, rehospitalisation, drop out and non‐ED psychopathology following treatment. Psychiatric comorbidity is known to increase risk for ED relapse following treatment (Sala et al. 2024). The fact that TE individuals were more likely to have continued concomitant symptoms following ED treatment could contribute to relapse and explain the higher rates of ED rehospitalisation in such individuals.

Our second aim was to understand how ED and PTSD symptoms impact improvements in each other. Our review builds on the findings of previous reviews (Convertino and Mendoza 2023; Day et al. 2024) by including studies that examine PTSD continuously as well as those that only included diagnostic status. Despite this broader inclusion criteria, results were largely consistent with previous reviews. Only four studies examined PTSD as a predictor or moderator of ED symptom improvement in an ED treatment, but three of the four found evidence that PTSD negatively impacts ED treatment outcomes. Despite this consensus, the small sample size only allows us to offer some tentative conclusions. As with TE, these studies seem to suggest that individuals with and without PTSD experience improvements in their ED symptoms by EOT and follow up. However, the majority of identified studies found evidence that patients may struggle to maintain any gains they experience in treatment once they are outside a structured programme, possibly due to the continuation of unresolved trauma symptoms that are triggered by environmental stressors and prompt the use of ED behaviours to cope. Actively addressing trauma‐related thoughts and emotions may be a necessary component of ED treatment for people with ED‐PTSD to ensure long term maintenance of gains.

Including a continuous measure of PTSD also allowed us to expand on previous reviews by including studies that examined ED as a predictor of PTSD symptom improvement. Our review identified only four studies that examined this relationship in a PTSD treatment, and two of these studies examined a PTSD‐SUD treatment. These studies indicate that EDs do interfere with PTSD treatment outcomes, which, again, supports a bidirectional relationship between ED‐PTSD. However, limited conclusions can be drawn from these few studies. More research is needed to examine how ED symptoms impact PTSD symptom improvement in treatment.

Our third aim was to examine how improvements in ED and PTSD symptoms might differ across treatment focus and modality. The studies included in this review reflect a wide array of ED and trauma‐focused approaches including CBT, psychodynamic, compassion focused, transdiagnostic, and integrative treatments. With few exceptions, all of the treatments showed significant improvements in ED, PTSD, and ED‐PTSD symptoms regardless of baseline trauma history, PTSD, or ED symptoms. Moreover, none of the eight RCTs that compared established treatments found a significant difference in symptom improvement between conditions. This suggests that individuals with trauma and EDs with or without PTSD can respond well to a variety of established and emerging approaches. Finally, results of this review overwhelmingly support the call made by many clinical researchers for concurrent or integrated treatments (Mitchell, Scioli, et al. 2021; Trim et al. 2018), and suggest that such treatments may result in broader improvements for individuals with comorbid ED‐PTSD than traditional single‐disorder treatments.

### 4.1 Strengths and Limitations

Strengths of reviewed studies include the fact that studies encompassed a wide range of ED diagnoses, treatment settings, and levels of care. However, few studies included patients with ARFID, and ethnic, racial and global diversity were limited across studies. Methodological quality of included studies also varied and there remains room for improvement. For instance, few studies used gold‐standard measures of PTSD or validated assessments of trauma exposure. Strengths of this review include a comprehensive search strategy and inclusion of predictors assessed both categorically (i.e., diagnosis) and continuously. However, few studies included effect sizes and we were unable to examine patterns across certain population (trauma type, age) or treatment (individual vs. group) characteristics.

### 4.2 Future Directions

Our review highlights several avenues for future research. First, the small number of studies identified in our review that examined the impact of ED on PTSD outcomes highlights that this is an existing gap in the literature. In order to inform understanding of the functional relationship between ED‐PTSD, more studies that examine how ED symptoms impact PTSD symptom improvement in PTSD treatments are needed. Second, given emerging research demonstrating high rates of PTSD associated with ARFID (MacDonald et al. 2024), studies examining ED‐PTSD populations should investigate how trauma and PTSD may be associated with clinical outcomes among this diagnostic group. Likewise lack of diversity is a well‐documented limitation of research on EDs (Egbert et al. 2022) and perpetuates biases in extant knowledge on how trauma and PTSD intersect with EDs across different sociocultural groups. Future research should rectify inequities in ethnic, racial and geographic diversity. Finally, studies that use dynamic and personalised longitudinal methods such as ecological momentary and idiographic network analyses to examine nuanced bidirectional associations between ED and PTSD during treatment would build on existing studies among non‐treatment seeking samples (e.g., Liebman et al. 2021; Nelson et al. 2022; Karr et al. 2013) and would be highly informative for theory and practice. Meta analyses that consolidate effect sizes of ED‐PTSD treatment outcomes across studies would also be highly beneficial to the field.

### 4.3 Conclusion

Results of this review suggest that PTSD symptoms, but not TE, interfere with ED treatment outcomes, and, likewise, that ED symptoms may interfere with PTSD symptom improvement. More specifically, those with ED‐PTSD do not experience worse improvement in treatment but may be more susceptible to treatment drop out and symptom relapse than either disorder alone. Findings also suggest that there are a number of promising established and emerging treatments for ED‐PTSD including several integrated and concurrent treatments. Treatments need to target the relationship between ED and PTSD symptoms and address long term strategies to reduce the likelihood of symptom relapse. A holistic approach to treating ED‐PTSD may offer the most lasting improvements in both conditions.

## Conflicts of Interest

The authors declare no conflicts of interest.

## Data Availability

Data sharing is not applicable to this article as no new data were created or analysed in this study.

## Endnotes

^1^
Cabelguen et al. (2023) did not describe the format of treatment and so are not included in treatment counts.
